# Stay-green traits to improve wheat adaptation in well-watered and water-limited environments

**DOI:** 10.1093/jxb/erw276

**Published:** 2016-07-21

**Authors:** John.T. Christopher, Mandy J. Christopher, Andrew K. Borrell, Susan Fletcher, Karine Chenu

**Affiliations:** ^1^The University of Queensland, Queensland Alliance for Agriculture and Food Innovation (QAAFI), Leslie Research Facility, PO Box 2282, Toowoomba, QLD 4350, Australia; ^2^Department of Agriculture and Fisheries Queensland, Leslie Research Facility, PO Box 2282, Toowoomba, QLD 4350, Australia; ^3^The University of Queensland, QAAFI, Hermitage Research Facility, 604 Yangan Road, Warwick, QLD 4370, Australia; ^4^The University of Queensland, QAAFI, 203 Tor St., Toowoomba, QLD 4350, Australia

**Keywords:** Crop adaptation, crop improvement, drought, genotype×environment interaction, leaf senescence, phenotyping, stay-green, water limitation, wheat.

## Abstract

A stay-green phenotype enables crops to retain green leaves longer after anthesis compared with senescent types, potentially improving yield. Measuring the normalized difference vegetative index (NDVI) during the whole senescence period allows quantification of component stay-green traits contributing to a stay-green phenotype. These objective and standardized traits can be compared across genotypes and environments. Traits examined include maximum NDVI near anthesis (Nmax), senescence rate (SR), a trait integrating senescence (SGint), plus time from anthesis to onset (OnS), mid-point (MidS), and near completion (EndS) of senescence. The correlation between stay-green traits and yield was studied in eight contrasting environments ranging from well watered to severely water limited. Environments were each classified into one of the four major drought environment types (ETs) previously identified for the Australian wheat cropping system. SGint, OnS, and MidS tended to have higher values in higher yielding environments for a given genotype, as well as for higher yielding genotypes within a given environment. Correlation between specific stay-green traits and yield varied with ET. In the studied population, SGint, OnS, and MidS strongly correlated with yield in three of the four ETs which included well-watered environments (0.43–0.86), but less so in environments with only moderate water-stress after anthesis (−0.03 to 0.31). In contrast, Nmax was most highly correlated with yield under moderate post-anthesis water stress (0.31–0.43). Selection for particular stay-green traits, combinations of traits, and/or molecular markers associated with the traits could enhance genetic progress toward stay-green wheats with higher, more stable yield in both well-watered and water-limited conditions.

## Highlight

Combining stay-green traits and environmental water-stress characterization, both standardized relative to anthesis, provides a powerful method to characterize and select for adaptation to well-watered and water-stressed environments.

## Introduction

Developing cultivars with superior adaptation to water-limited environments has been impeded by complex interactions between genotype and environment (G×E), leading to changes in the yield rankings of genotypes in different water-limited environments (Cooper *et al.*, 2001; Richards *et al.*, 2002). To improve the rate of yield gain in the face of G×E, researchers have sought physiological and morphological traits linked with high yield in the target population of environments (TPE) that are less susceptible to environmental influences than yield *per se* (Jackson *et al.*, 1996; Hammer *et al.*, 2002, 2005; Tardieu 2003; Casadebaig *et al.*, 2016). The stay-green phenotype has been linked to improved yield stability in a number of cereal crop species including wheat and sorghum, particularly under terminal drought stress (recently reviewed in Gregersen *et al.*, 2013). Plants exhibiting the stay-green phenotype are able to maintain green leaf area for longer after anthesis than senescent lines, allowing maintenance of photosynthesis for longer during the grain-filling period (Thomas and Smart, 1993; Thomas and Howarth, 2000). Thus, selection for stay-green has been targeted to improve crop adaptation to water-stressed environments in a number of crops including sorghum and wheat (Christopher *et al.*, 2008, 2014; Borrell *et al.*, 2012, 2014*a*, *b*; Jordan *et al.*, 2012; Lopes and Reynolds, 2012; Gregersen *et al.*, 2013).

The stay-green phenotype has long been recognized as having potential for crop improvement (Thomas and Smart, 1993). This phenotype can be either functional, where photosynthesis and accumulation of assimilates to harvested tissues are prolonged, or non-functional, where plants appear green but there is no benefit in terms of yield (e.g. due to a lesion in the chlorophyll recycling process, or disrupted transfer of nitrogen from leaf to grain). Only functional stay-green is of interest for crop improvement. Functional stay-green can be achieved by varying leaf-greenness dynamics in a number of different ways (Thomas and Howarth, 2000). Plants may be greener around anthesis before the onset of senescence, commence senescence later, or senesce more slowly (Thomas and Howarth, 2000; Harris *et al.*, 2007; Christopher *et al.*, 2014).

Stay-green has previously been assessed in the field using various techniques. Rapid evaluation by visual assessments have been performed by rating whole-plant senescence (Rosenow *et al.*, 1983; Henzell *et al.* , 1992; Jordan *et al.*, 2012), or assessing green leaf number per culm (Haussman et al., 1999), the greenness of all fertile shoots (Foulkes *et al.*, 2007), or greenness of the flag leaf and peduncle (Joshi *et al.*, 2007). More objective measures of greenness have been taken for individual leaves with the Minolta SPAD meter (Borrell *et al.*, 1996; Harris *et al.*, 2007; Christopher *et al.*, 2008) and, more recently, the canopy with normalized difference vegetative index (NDVI)-based methods (e.g. Lopez and Reynolds, 2012; Christopher *et al.*, 2014). Those methods commonly relied on measurements from one or a few time points late in the crop cycle, with linear regressions fitted to model senescence dynamics (e.g. Harris *et al.*, 2007; Lopes and Reynolds, 2012). However, traits such as onset of leaf senescence vary between genotypes, and the date of measurements can impact the results (Christopher *et al.*, 2014). In addition, the dynamics of senescence appear to follow a non-linear pattern (Borrell *et al.*, 2000*a*; Christopher *et al.*, 2008, 2014; Vijayalakshmi *et al.*, 2010).

A method has recently been proposed to assess quantitative, component stay-green traits in field trials (Christopher *et al.*, 2014). The dynamics of canopy senescence were shown to fit closely a logistic model fitted to periodic NDVI measured using the NTech Greenseeker^®^. This information was then used to estimate a number of component stay-green traits including the timing from anthesis to (i) senescence onset (OnS); (ii) mid-senescence (MidS); and (iii) near completed senescence (EndS); as well as (iv) the initial NDVI level (near anthesis; Nmax); (v) an indicator of the senescence rate (SR); and (vi) a parameter derived by integrating NDVI over the senescence period that provides a measure somewhat analogous to green leaf area duration (SGint; Table 1). This approach provides a more detailed understanding of genotypic variation in stay-green phenotype by examining contributing traits during the whole senescence period for each genotype. Traits are estimated based on objective NDVI measurements which are fitted to a logistical model standardized to thermal time with respect to anthesis for each genotype. This enables genotypes to be compared both within and across environments. The method also allows hundreds of genotypes to be characterized in multiple environments, allowing investigations of G×E and characterization of populations for genetic studies.

**Table 1.** Abbreviations and descriptions of stay-green traits Stay-green traits were estimated for each plot of each genotype from a logistic function fitted to NDVI field data centred at anthesis.

Abbreviation	Stay-green trait	Description
Nmax	Maximum leaf greenness	Maximum NDVI value usually near anthesis
OnS^*b*^	Onset of leaf senescence	Thermal time from anthesis to 90% of *N*_*green max*_^*a*^
MidS^*b*^	Mid-point of leaf senescence	Thermal time from anthesis to 50% of *N*_*green max*_^*a*^
EndS^*b*^	Near completion of leaf senescence	Thermal time from anthesis to 10% of *N*_*green max*_^*a*^
SR	Indicator of the rate of senescence	Indicator of the rate of NDVI decrease at MidS
SGint	Stay-green integral (senescence integral)	Cumulative NDVI from anthesis to after senescence completion at 1500 ^o^Cd after anthesis.

^*a*^ *N*_*green max*_, difference between the Nmax and the final NDVI value at 1500 ^o^Cd.

^*b*^ OnS, MidS, and EndS have previously been labelled TFN90, TFN50, and TFN10, respectively, in Christopher *et al.* (2014)

Recent advances have also occurred in our understanding of the patterns of seasonal water deficit in the target population of environments (TPE) of Australian rain-fed wheat crops (Chenu *et al.*, 2011, 2013). Changes in the timing of water deficit with respect to crop development lead to different impacts of water-stress by affecting different processes. Chenu *et al.* (2013) determined that seasonal water-stress patterns encountered by Australian wheat crops can be classified into four main environment types (ETs), dependent upon the timing and severity of water-stress from least stressed ET1 to the most stressed ET4. Knowledge of the classification of environments encountered in multienvironment field trials can aid breeders, in particular, by providing information about whether specific trials are more or less representative of the environments encountered in the TPE (Chenu *et al.*, 2011; Chenu, 2015). Thus, breeders could potentially ‘weight’ trial results according to the relevance of the particular ET in the TPE. They can also aid researchers to choose and/or manipulate environmental conditions to generate environments that will be representative of the TPE; and to interpret the value of traits, including stay-green traits, across environments (Rebetzke *et al.*, 2013; Chenu, 2015).

The aim of the current study was to determine the potential of the recently proposed component stay-green traits for crop improvement. Can stay-green traits be useful to select for high-yielding genotypes in a broad range of water-stress environments or, conversely, for adaptation to specific classes of water-stress environments? What is the magnitude of the effect of individual stay-green traits in different environments? A mapping population of doubled-haploid lines segregating for stay-green traits was studied over 3 years in a total of eight trials where rainfall, irrigation, and/or rain-out shelters were employed to generate a wide range of water-stress environments encountered in the TPE. Correlations between stay-green traits and yield, and the apparent effect of traits on yield, were examined to identify traits suitable for selection of high-yielding, stay-green genotypes.

## Materials and methods

### Plant material

To reduce variation for height and maturity date, 184 lines were selected from a much larger doubled-haploid population derived from the bread wheat (*Triticum aestivum*) cultivars SeriM82 and Hartog (Christopher *et al.*, 2013, 2014). The parental lines contrast for yield and stay-green. SeriM82 is a high-yielding drought-tolerant, stay-green line (Sivapalan *et al.*, 2000, 2001; Olivares-Villegas *et al.*, 2007), while Hartog is a senescent cultivar adapted to subtropical Australia (Manschadi *et al.*, 2006, 2010; Christopher *et al.*, 2008).

### Field trials

Field trials were established during three seasons (2010, 2011, and 2012) at three sites in southern Queensland, Australia: Gatton (GAT: 27.54^o^S, 152.34^o^ E, 89 m a.s.l.) in 2010, and Kingsthorpe (KTP: 27.51°S, 151.78°E; 442 m a.s.l.) and Warwick (WAR: 28.21^o^S, 152.10^o^E, 480 m a.s.l.) in 2011 and 2012. Crops were grown either in natural rain-fed conditions (‘rf’ treatment), in irrigated conditions (‘ir’) where irrigation was used to ensure little water-stress throughout the season, or under a rain-out shelter (‘ro’) which excluded rainfall from anthesis onwards. Some rain-fed treatments were also watered to near field capacity immediately after sowing to ensure uniform establishment. Experiment names are derived from a combination of location, year, and treatment such that the rain-fed trial at Warwick in 2011 is designated ‘WAR11rf’ (Table 2). Heavy, alkaline cracking clay soils with high water-holding capacity predominate at all sites.

**Table 2.** Trial identifier (Trial ID), sowing date, days from sowing to anthesis for the reference parent Hartog (DTA), days from sowing to maturity of Hartog (DTM), plant-available soil water capacity (PAWC, mm), plant-available soil water at sowing (PAW, mm), irrigation at sowing (Irri init, mm), irrigation immediately prior to anthesis (Irri anth, mm), cumulative in-crop rainfall (ICR, mm), water potentially available (WPA), average daily maximum temperature from sowing to maturity (Avg Temp, ^o^C), cumulative radiation from sowing to maturity (Cum Radn, MJ m^−**2**^), number of genotypes tested (No. genos), environment mean yield (Yld, g m^−2^), and environment type as depicted in Fig. 1 (ET)

Trial ID^*a*^	Sowing date	DTA	DTM	PAWC	PAW	Irri init	Irri anth	ICR	WPA^*b*^	Avg Temp	Cum Radn	No. genos	Yld	ET
**GAT10ir**	26 May	94	134	285	185	25	42	216	468	15.7	1808	143	464	**ET1**
**GAT10rf**	26 May	89	130	285	185	25	−	214	424	15.6	1741	101	498	**ET1**
**WAR11rf**	24 June	98	141	258	258	−	−	187	445	13.2	2447	183	567	**ET1**
**GAT11rf**	9 June	89	131	312	312	25	−	157	494	15.0	2157	151	659	**ET2**
**KTP11rf**	10 June	107	149	290	290	−	−	188	478	13.2	2543	182	579	**ET2**
**GAT12rf**	10 July	79	115	285	284	−	−	95	379	16.8	2202	191	442	**ET3**
**WAR12rf**	22 June	105	144	295	255	−	−	109	364	13.8	2649	189	415	**ET3**
**WAR12ro**	22 June	96	136	216	176	−	−	0	176	13.5	2485	76	208	**ET4**

^*a*^ Trial identifiers indicate the sites as Gatton (GAT), Kingsthorpe (KTP), or Warwick (WAR); the year from 2010 to 2012; and the treatment as irrigated (ir), rain-fed (rf), or rain-out shelter (ro).

^*b*^ Water potentially available (WPA)=PAW+Irri init+Irri anth+ICR.

Crops were sown in 2 m×6 m plots with a row spacing of 25cm and a target population density of 100 plants m^−2^. Soil tests were performed prior to planting to estimate parasitic nematode densities as well as soil N and P levels. Nematode densities were below known damage thresholds for sensitive wheat cultivars. Non-limiting levels of nutrients were applied using 120kg ha^−1^ urea prior to sowing and 40kg ha^−1^ of Starter Z^®^ containing 10.5% N, 19.5% P, 2.2% S, and 2.2% Zn at sowing. Weeds and diseases were controlled as necessary.

### Trial design and statistical analysis

All trials were designed as partially replicated row–column experiments (GAT10rf 39%, GAT10ir 38%, WAR11rf 38%, GAT11rf 37%, KTP11rf 42%, WAR12rf 32%, GAT12rf 36%, and WAR12ro 21%; Cullis *et al.*, 2006). The designs included an underlying component for autocorrelation in the column and row direction, and entries were latinized along rows and columns. All designs were generated using ‘lmmdesign’ (Butler *et al.*, 2008). Up to 184 SeriM82×Hartog double-haploids plus the parents were tested in each trial (Table 2).

The statistical analysis comprised a set of individual analyses and a set of bivariate analyses. Individual analyses were performed for each stay-green trait and yield in each trial. A linear mixed model was fitted to each separate data set. Each model included a random term for replicate blocks, a separable autoregressive structure for both rows and columns at the residual level, and terms to account for spatial field trend following the method of Gilmour *et al.* (1999). Genotypes were fitted as random to estimate the genetic effects of each trait within each trial. Best linear unbiased predictors (BLUPs) were calculated from each separate analysis. The BLUPs from each individual trial analysis of each trait were applied to a principal components analysis (PCA) using the ‘princomp’ function in the R software package ‘stats’ (R Development Core Team, 2013). To standardize the data across traits, the PCA was applied to a correlation matrix of the traits, implemented through the ‘cor’ command in princomp. This analysis projects the data onto a reduced set of components, where the first two components explain the majority of the variability in the data. The results were summarized as a biplot where scores for each genotype and the loading for each trait are plotted for these first two components (Gabriel, 1971).

A series of bivariate linear mixed models were performed to estimate the genetic variance of the stay-green trait, the genetic variance of yield, and the correlation between the two at each trial, as previously described in Christopher *et al.* (2014). Each model included the relevant random and spatial terms established in the individual trial analyses. The slope of the genetic regression was calculated between yield and each trait using the estimated genetic variances and correlation. An approximate standard error for each slope was calculated using Taylor expansion. The genetic correlations provide a measure of the strength of the agreement in genotype rankings between yield and each trait. The genetic regression provided a predictive quantity of the potential for the rate of change in yield based on the unit change in the trait (within the range of the data).

Individual and bivariate analyses were performed in ASReml-R (Butler *et al.*, 2009) using R software. Best linear unbiased estimates (BLUEs) were also calculated.

### Characterization of the water-stress environments

**Fig. 1.**
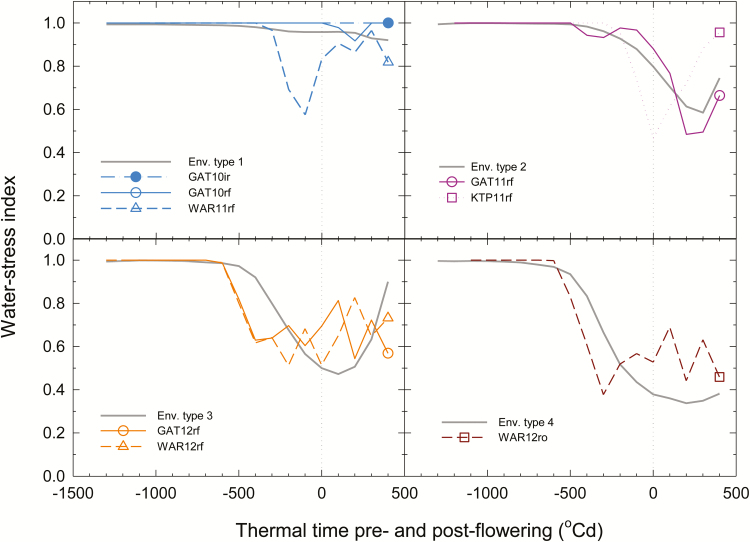
Seasonal crop water-stress patterns indicating the ratio of available water to potential transpiration demand (water-stress index) plotted against thermal time relative to anthesis of the reference genotype Hartog at each of eight trial environments. An irrigated trial at Gatton in 2010 (GAT10ir; ET1), and rain-fed trials at Gatton in 2010 (GAT10rf; ET1), Warwick in 2011 (WAR11rf; ET1), Gatton in 2011 (GAT11rf; ET2), Kingsthorpe in 2011 (KRP11rf; ET2), Gatton in 2012 (GAT12rf; ET3), and Warwick in 2012 (WAR12rf; ET3), as well as a trial with rain excluded using a rain-out shelter at Warwick in 2012 (WAR12ro; ET4). Thick grey lines represent the stress patterns for each of the main environment types (ETs) from the target population of environments as described by Chenu *et al.* (2013). Anthesis is indicated by the vertical dotted lines.

Crops in each trial were simulated with the computer crop simulation model, the Agricultural Production Systems sIMulator APSIM-wheat v7.0 (Holzworth *et al.*, 2014), using climatic and soil data collected at each site. The water deficit patterns experienced by the parent cultivar Hartog were determined for each trial based on the water-stress index computed by APSIM. The water-stress index corresponds to a water supply/demand ratio that integrates the crop demand and the water available to the roots. A ratio of 1 indicates no water-stress, while a ratio of 0 corresponds to a full stress, with no water available to the crop (Chenu *et al.*, 2013). To ensure best estimation of the stress pattern for each trial, simulated anthesis dates from APSIM were adjusted to concur with the observed date of anthesis, by adapting the thermal time duration between emergence and floral initiation. Simulations of yield closely or slightly underpredicted grain yield (Supplemetnary Fig. S1 at *JXB* online), suggesting that transpiration patterns and water-deficit responses were well approximated. Data from all trials were centred at anthesis, and averaged every 100degree Celsius days (^o^Cd) between emergence and 450 ^o^Cd after anthesis. The trials were classified according to their similarity to the four previously identified main ETs from the Australian TPE (Fig. 1; Chenu *et al.*, 2011, 2013).

The four ETs are defined as ET1–ET4, which rank roughly in ascending order of water-stress such that ET1 represents environments where crops experience little or no water-stress, ET2 where crops experience moderate to severe water-stress of short duration mainly after anthesis, ET3 where crops mainly experience moderate to severe water-stress from the lead up to anthesis and the early grain-filling period, and ET4 where severe water-stress leading up to anthesis is not relieved (Chenu *et al.*, 2013).

### Crop measurements

Emergence counts were taken to ensure plots were well established. For each plot, Zadoks stages were recorded weekly to determine anthesis date (Zadoks code 65, Z65; Zadoks *et al.*, 1974). NDVI was measured weekly for each plot starting from awn emergence (Z49) until after maturity using a hand-held Greenseeker model 505 (NTech Industries, Ukiah, CA, USA). At the end of the experiment, grain was harvested using a small plot harvester to estimate yield expressed in g m^−2^. The few plots affected by disease were removed from the analysis. Crop height was measured to the top spikelet of the ear, not including the awns, near the time of anthesis.

### Estimation of stay-green traits

Stay-green traits (Table 1) were estimated from a logistic function fitted to NDVI data centred at anthesis for each plot (for more details, see Christopher *et al.*, 2014).

## Results

### Each of the four major water-stress environment types in the TPE were represented in the multienvironment trials

Using a combination of natural environmental variation and applied treatments, each of the major water-stress ETs described for wheat crops in the Australian TPE were sampled (Table 2; Fig. 1; Chenu *et al.*, 2013).

All sites had a significant amount of plant-available water (PAW) stored in the soil prior to sowing (Table 2). In-crop rainfall varied from 0mm at WAR12ro to 216mm at GAT10ir. The environments produced a wide range of yields, with trial mean yields for all genotypes ranging from 208g m^−2^ in the rain-out shelter at WAR12ro to 659g m^−2^ at GAT11rf (Table 2).

The seasonal water-stress patterns for each of the eight environments were classified into one of the four water-stress ETs previously identified (Table 2; Fig. 1). GAT10ir, GAT10rf, and WAR11rf only exhibited minor water deficits throughout the season, and so were classified in ET1 (Fig. 1). For crops at GAT11rf and KTP11rf, water deficit built up to reach a maximum near or following anthesis, but this stress was relieved by rainfall soon thereafter, allowing grain filling to finish with little water-stress, corresponding to ET2 (Fig. 1). Greater stress affected crops at GAT12rf and WAR12rf, where water deficit became moderate to severe well before anthesis and continued well into the grain-filling period (ET3; Fig. 1). The most severe water-stress was observed at WAR12ro, where a severe water deficit from before anthesis continued through to maturity (ET4; Fig. 1).

### Yield tended to decrease with increasing water-stress from ET1 to ET4

**Fig. 2.**
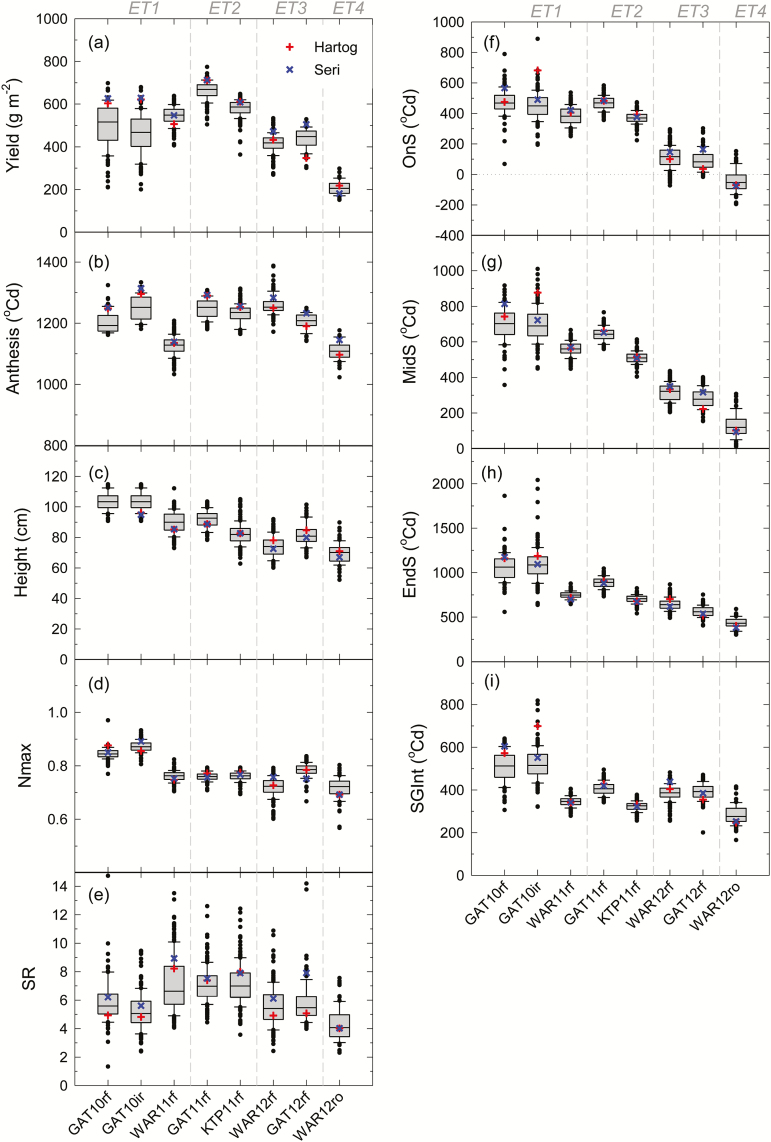
Range of genetic means for (a) yield, (b) thermal time from sowing to anthesis, (c) plant height, (d) maximum NDVI around anthesis (Nmax), (e) senescence rate (SR); thermal time from anthesis to (f) senescence onset (OnS), (g) mid-senescence (MidS), (h) senescence near completion (EndS), and (i) stay-green integral (SGint) for eight trials in southern Queensland Australia. An irrigated trial at Gatton in 2010 (GAT10ir; ET1), and rain-fed trials at Gatton in 2010 (GAT10rf; ET1), Warwick in 2011 (WAR11rf; ET1), Gatton in 2011 (GAT11rf; ET2), Kingsthorpe in 2011 (KRP11rf; ET2), Gatton in 2012 (GAT12rf; ET3), and Warwick in 2012 (WAR12rf; ET3), as well a trial with rain excluded using a rain-out shelter at Warwick in 2012 (WAR12ro; ET4). Data are best linear unbiased predictors (BLUEs). Individual BLUEs are indicated for the parent lines Hartog (+) and Seri (×). For the boxplots, the middle line of the box represents the median, the upper and lower edges represent the 75th and 25th percentiles; the whiskers the 10th and 90th percentiles; and the dots outside the whiskers represent individual values outside this range. Details in regards to environment types (ET1–ET4) are illustrated in Fig. 1. (This figure is available in colour at *JXB* online.)

As anticipated, water availability was a major yield constraint in the studied environments. There was a general trend to decreased yield as the water-stress pattern became more severe from the least stressed ET1 through to the most stressed ET4 (Table 2; Fig. 2). Although environment mean grain yield of the eight environments was not well correlated with in-crop rainfall (Table 2), it was strongly, positively correlated with the amount of potentially available moisture, which was estimated as PAW at sowing plus in-crop rainfall and irrigation, particularly in 2011–2012 trials (Table 2).

Other factors probably influenced yield in certain environments. For example, environments at Gatton 2010 had the highest in-crop rainfall and potentially available water, but yield was lower than environments in 2011 (Table 2). The high in-crop rainfall at Gatton 2010 was accompanied by increased cloud cover, which decreased incident radiation. Cumulative radiation for environments at GAT10ir (1808 MJ m^–2^) and GAT10rf (1741 MJ m^−2^) were well below those for other environments, which ranged from 2157 MJ m^−2^ to 2649 MJ m^−2^ (Table 2), particularly during the grain-filling period (Supplementary Fig. S2b).

In addition, crops in some environments with a weekly mean of the daily maximum temperatures reaching or exceeding 30 ^o^C probably experienced heat stress around anthesis and during the grain-filling period (Supplementary Fig. S2). Prolonged temperatures above 30 ^o^C pre- and post-anthesis are known to affect wheat productivity (e.g. Tashiro and Wardlow, 1990; Reynolds *et al.*, 2001). Heat stress could potentially have affected yield in all environments classified in ET3 and ET4, as well as GAT11rf in ET2 (Supplementary Fig. S2, solid lines). Environments in ET1 as well as KTP11rf in ET2 did not reach these temperatures during the early- or mid-grain filling period, or reached them only late in development when starch deposition would have been nearly complete (Supplementary Fig. S2). Thus, heat stress is less likely to have affected yield in these environments.

Yield rankings for genotypes varied between environments, indicating crossover G×E. Genetic correlations between pairs of environments varied from −0.45 to +0.87, and the mean correlation for all eight environments was +0.3. In the overall analysis, the variance component due to G×E was 1.6 times greater than that for genotype. These results are not uncommon for multienvironment trials in the Australian TPE and highlight the need to seek adaptation indicators with higher heritability and lower G×E than yield *per se.*

### Stay-green traits were strongly affected by changes in water-stress between environments

Variation between genotypes for yield tended to decrease with increased water-stress and lower yield from ET1 to ET4 (Fig. 2a). The trend towards lower yield with increasing water-stress was accompanied by a marked shortening of the period from anthesis to the onset of senescence (OnS), mid-point of senescence (MidS), and near completion of senescence (EndS; Fig. 2f–h). Smaller relative decreases from ET1 to ET4 were also observed for the indicator of the maximum senescence rate (SR; except for the two light-constrained environments of GAT10), the maximum NDVI near anthesis (Nmax), and the stay-green integral (SGint).

Water-stress tended to shorten the thermal time period from sowing to anthesis, but only in some environments (Fig. 2b). There was a clear trend towards shorter plants in more water-stressed environments, ranging from an environment mean of 104cm at GAT10ir in ET1 to 70cm at WAR12ro in ET4 (Fig. 2c).

### Higher values of Nmax, OnS, MidS, EndS, and SGint are correlated with higher yielding environments

**Fig. 3.**
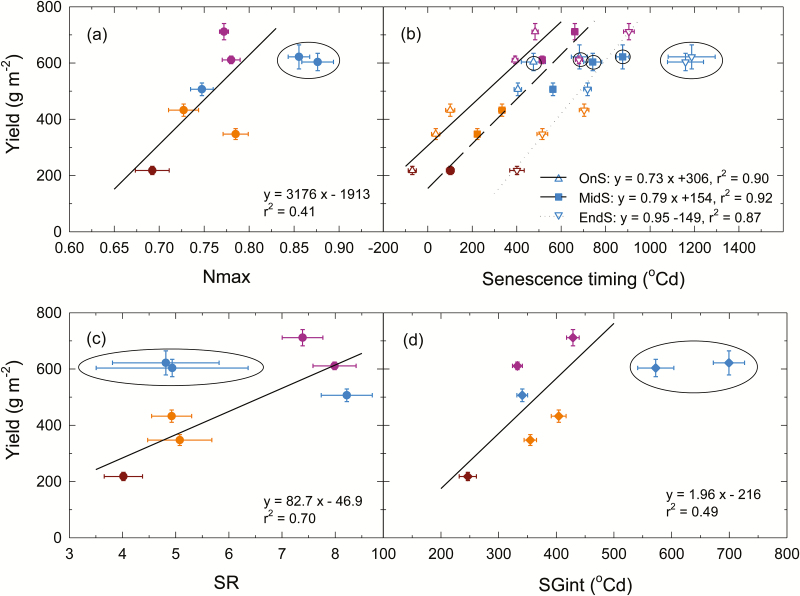
Linear regression between BLUES of standard cultivar Hartog for the stay-green traits (a) maximum NDVI around anthesis (Nmax); (b) thermal time from anthesis to senescence onset (OnS), mid-senescence (MidS), and senescence near completion (EndS); (c) indicator of maximum senescence rate (SR); and (d) stay-green integral (SGint) against mean yield in eight environments in subtropical Australia. Symbol colours indicate different environment types (ET): ET1 (blue), ET2 (mauve), ET3 (orange), and ET4 (brown) as in Fig 5. Error bars indicate the SE of each mean. Irrigated and rain-fed trials at Gatton in 2010 (GAT10ir and GAT10rf), which had lower yield probably due to radiation limitation, were excluded from the fitted regressions (blue points circled in black).

There is a clear positive correlation between environment mean yield of the standard genotype Hartog and higher environment mean values for Nmax, OnS, MidS, EndS, SR, and SGint (Fig. 3), with less water-stressed environments generally having higher yield and higher stay-green trait values in the order ET1–ET2> ET3> ET4 (Figs 2, 3). The slope of these regression lines provides an estimate of the apparent effect of stay-green traits on the yield of Hartog across environments. For example, yield increases by 0.73g m^−2^ for each ^o^Cd increase in OnS (Fig. 3b). If we consider a typical day (18 ^o^Cd) during the early grain-filling period in the TPE, this corresponds to an ~13g m^−2^ increase for a 1 d delay in senescence onset, representing a 3.2% yield increase for a trial with a mean yield of 400g m^−2^. Change in MidS had an impact of a similar order of magnitude on yield, and EndS slightly higher (Fig. 3b). Thus, delays of only a few days in OnS, MidS, and EndS in the different environments were correlated with considerable increases in the yield of Hartog. Substantial impacts of Nmax, SR, and SGint were also observed (Fig. 3a, c, d), but it is important to remember that these stay-green traits can be closely correlated to each other. Somewhat counter-intuitively, the positive correlation between SR and yield in several environments indicates that the higher yield is correlated with a faster rate, as discussed below.

### Genetic correlations between stay-green traits and yield varied with the water-stress environment type

**Fig. 4.**
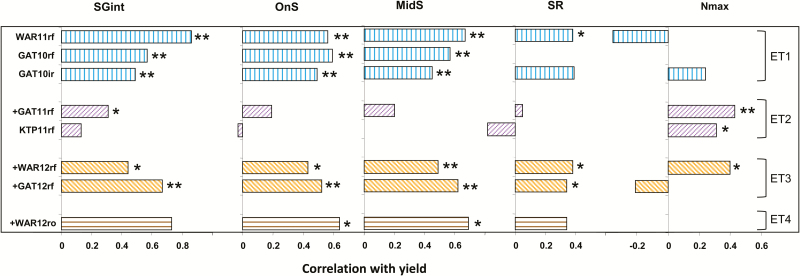
Genetic correlations between yield and stay-green traits in eight field environments in southern Queensland, Australia. Stay-green traits, maximum NDVI around anthesis (Nmax), indicator of maximum senescence rate (SR); stay-green integral (SGint), thermal time from anthesis to senescence onset (OnS), and mid-senescence (MidS) are defined in Table 1, and environment types (ET1–ET4), as indicated at the right hand side, are illustrated in Fig. 1. Significance levels for bivariate comparisons are indicated at the levels *P*≤0.01 (**), *P*≤0.05 (*). A bar is absent when there was little variation for the trait in the particular environment or when the correlation between the trait and yield was close to zero. Slopes of regressions fitted to these correlations are given in Table 3. (This figure is available in colour at *JXB* online.)

High correlations between stay-green traits and yield were observed across genotypes within most trials (Fig. 4). The degree of genetic correlation varied with the water-stress ET. The timings of senescence onset (OnS) and of the mid-point of senescence (MidS) were significantly correlated with yield in ET1, ET3, and ET4 (*P*≤0.05 or ≤0.01) but not in ET2 (*P*>0.05; Fig. 4). The SGint was also significantly correlated with yield in ET1, in one ET2 (GAT11rf), and in ET3 (*P*≤0.05). In ET4, the correlation between SGint and yield was close (0.73), but not statistically significant (*P*>0.05). The lower number of genotypes tested in ET4 (76) and reduced yield variation due to severe stress (Fig. 2a) may have reduced the likelihood of detecting a significant correlation in this environment. Finally, the genetic correlation between mean estimated EndS and yield was significant for only two environments, GAT10rf and GAT12rf (0.42 and 0.39, respectively; *P*≤0.05; not shown).

In contrast to OnS, MidS, and SGint, correlations between yield and SR or Nmax were more varied across water-stress environments. The maximum leaf canopy greenness (Nmax) was correlated with yield for both environments classified as ET2 and one in ET3 (WAR12rf). The SR was significantly correlated with yield in both ET3 environments, but in other ETs only at WAR11rf (ET1). Height and time to anthesis were not significantly correlated with yield in most environments, probably reflecting the previous selection of the population to reduce variation for these traits (Christopher *et al.*, 2013). The range of BLUPs for anthesis dates among the doubled-haploids was from 4.0 d at GAT12rf to 4.6 d at GAT10rf, 4.9 d at HRS12rf, 7.1 d at GAT11rf, 7.3 d at KTP1rf, 8.0 d at GAT10ir, and 10.7 d at WAR11rf. However, yield was significantly positively correlated with time to anthesis at one of the radiation-limited environments (GAT10rf, *P*<0.05) and significantly negatively correlated with height at the other radiation-limited environment (GAT10ir, *P*<0.05) and at one ET2 environment (KTP11rf, *P*<0.001). Including these traits as covariates in the statistical model for the yield prediction (BLUPs) did not significantly affect the results for any environment, so the analysis without covariates was used.

No clear pattern in the genetic correlations between stay-green traits and yield was observed in sites affected by heat stress (i.e. all ET3 and ET4 environments, as well as GAT11rf in ET2) compared with others (Fig. 4; Supplementary Fig. S2). Similarly, there was no clear difference in the pattern of genetic correlations for the potentially radiation-limited environments at GAT10ir and GAT10rf compared with the other likely non-radiation-limited environments (Fig. 4; Supplementary Fig. S2).

### Greater genotype mean values for Nmax, OnS, MidS, EndS, and SGint are correlated with higher yield within environments

The ability to retain green leaf area had significant effects on yield of individual genotypes which varied with environment type. The slope of regression lines used to estimate the correlations between stay-green traits and yield at each site (Fig. 4) give an indication of the magnitude of the apparent effect of stay-green traits on yield in various environments (Table 3). For example, an increase in OnS by 1 ^o^Cd led to a yield increase from little or none at KTP11rf up to 0.96g m^−2^ at GAT10rf (Table 3). For a typical day of 18 ^o^Cd during early grain filling, this equates to a yield increase of 17.3g m^−2^ d^−1^ at GAT10rf, representing ~3.5% of the trial mean yield of 498g m^−2^ for each day’s delay in OnS. Similarly, for MidS, the gains range up to 0.66g m^−2^ per ^o^Cd delay at GAT10rf, equating to 9.9g m^−2^ for each 18 ^o^Cd day, or ~2.4% of the trial mean yield. Thus, a delay of just a few days in OnS or MidS, or an increase in Nmax or SGint was associated with considerable yield differences between genotypes. The apparent magnitude of impact for delayed OnS is similar to that observed for changes between environments in the mean yield of Hartog discussed above (Fig. 3).

**Table 3.** Slope and the SE of the slope of bivariate regressions between yield and stay-green traits Nmax, OnS, MidS, SR, and SGint for the eight studied environments

	Nmax		OnS		MidS		SR		SGint		ET
	Slope	SE	Slope	SE	Slope	SE	Slope	SE	Slope	SE	
**GAT10ir**	17.74	1.57	0.49	0.16	0.38	0.10	0.41	0.39	0.53	0.15	ET1
**GAT10rf**			0.96	0.40	0.66	0.24			0.84	0.19	ET1
**WAR11rf**	−20.08	7.01	0.34	0.11	0.60	0.16	0.07	0.03	2.25	1.13	ET1
**GAT11rf**	12.65	0.39	0.35	0.18	0.24	0.17	0.02	0.06	0.58	0.19	ET2
**KTP11rf**	9.79	0.43	−0.04	0.18	0.00	0.28	−0.06	0.04	0.35	0.47	ET2
**WAR12rf**	4.56	0.16	0.26	0.09	0.44	0.13	0.11	0.05	0.54	0.17	ET3
**GAT12rf**	−9.47	1.94	0.37	0.08	0.49	0.09	0.09	0.38	0.92	0.23	ET3
**WAR12ro**			0.15	0.07	0.16	0.07	0.05	0.07	0.29	0.18	ET4

The apparent effects on yield of SGint, OnS, and MidS were greatest in ET1 and ET3. They were low in the mild, late-stressed ET2. They were also low in the worst-stressed ET4, possibly resulting from reduced variation in yield in this environment (Fig. 2). There seems to have been little effect of SR on yield in most environments, except GAT10ir (Table 3). The effect of Nmax was varied, ranging from negative to positive in ET1 and ET3. However, there was a consistent positive effect of Nmax on yield in the mild, later-stressed ET2.

**Fig. 5.**
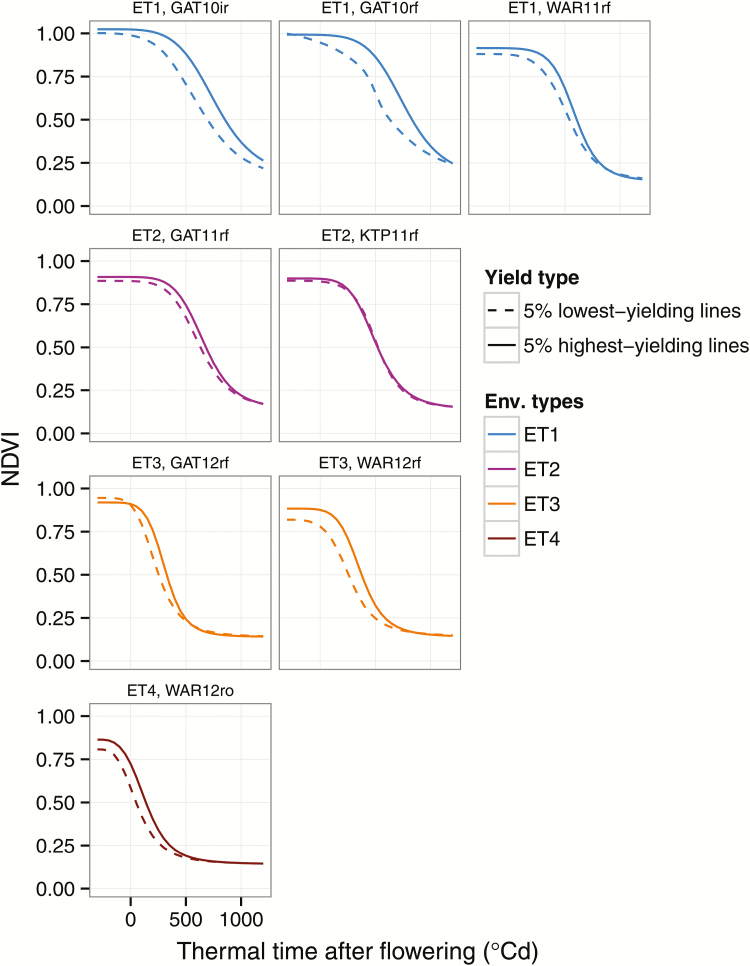
Logistic regressions of mean normalized difference vegetative index (NDVI) of the highest yielding 5% of genotypes (solid lines) and the lowest yielding 5% of genotypes (dashed lines) at the eight studied environments plotted over thermal time relative to anthesis. Note that differences between high- and low-yielding groups for anthesis were small (from −35 to +38 ^o^Cd) and non-significant for all environments (Student’s *t*-test; *P* > 0.05).

To examine further the differences in stay-green traits among genotypes within environments, we plotted the average NDVI of the 5% highest yielding genotypes against the 5% lowest yielding genotypes for each environment (Fig. 5). Clear differences were observed between the highest and lowest yielding genotypes in most environments, with the higher yielding genotypes retaining green leaf area for longer (Fig. 5). The differences appear greatest in the two radiation-limited ET1 environments at Gatton 2010 (GAT10ir, GAT10rf; Fig. 5), where variation for yield was also greatest (Fig. 2). The smallest differences in NDVI between the high- and low-yielding groups were in the two ET2 environments (GAT11rf and KTP11rf, Fig. 5), where significant correlation between yield and stay-green traits OnS, MidS, and SR was mostly absent (Fig. 3). These results are in accordance with the genetic correlations observed between yield and stay-green traits (Fig. 4).

The graphs in Fig. 5 also illustrate why the correlation between SR and yield is positive in some environments, which could at first seem counter-intuitive (Fig. 3). In all environments, the high-yielding genotypes commence senescence later than the low yielders, but NDVI difference narrows as senescence progresses, such that they attain full senescence at a similar time after anthesis (Fig. 5). Commencing senescence later, but completing senescence at a similar time, requires an increased rate of senescence but it results in an increase in overall green leaf area retention (measured by SGint) and increased yield.

It is unlikely that yield contrast between high- and low-yielding groups result from differences in anthesis date. Differences between groups in the mean period from sowing to anthesis were small (ranging from −35 ^o^Cd to +38 ^o^Cd) and non-significant for all environments (Student’s *t*-test; *P*>0.05).

### Relationships between stay-green traits varied between environments

**Fig. 6.**
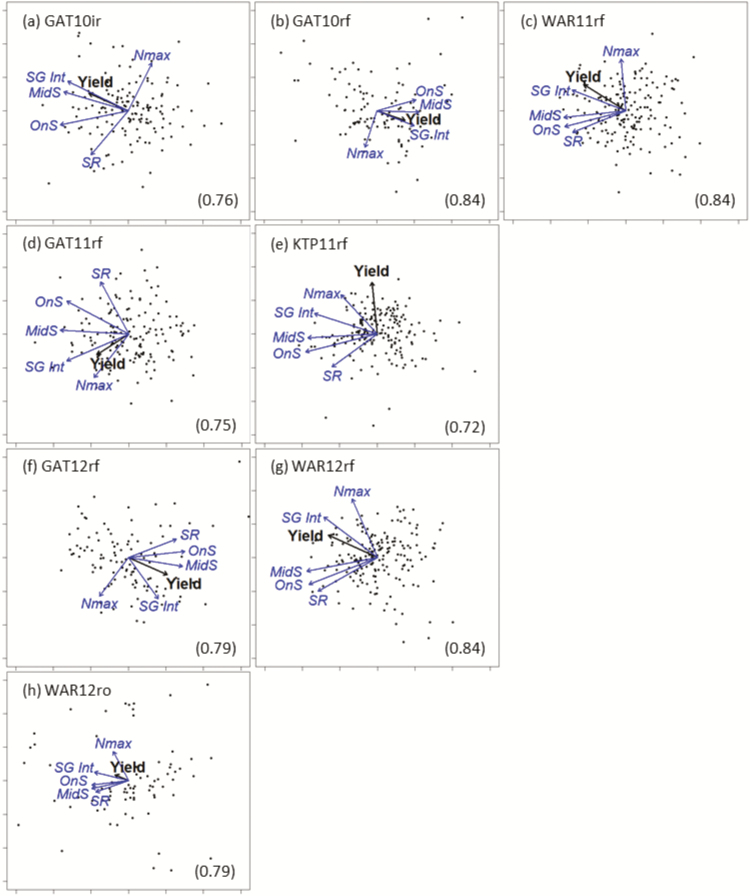
Biplots of results from principal components analyses (PCA) indicating correlations between yield and stay-green traits for (a) an irrigated trial at Gatton in 2010 (GAT10ir; ET1), and rain-fed trials in (b) Gatton in 2010 (GAT10rf; ET1), (c) Warwick in 2011 (WAR11rf; ET1), (d) Gatton in 2011 (GAT11rf; ET2), (e) Kingsthorpe in 2011 (KRP11rf; ET2), (f) Gatton in 2012 (GAT12rf; ET3), and (g) Warwick in 2012 (WAR12rf; ET3), as well as a trial with rain excluded using a rain-out shelter at (h) Warwick in 2012 (WAR12ro; ET4). The direction and magnitude of the vectors represent the effects, in relation to the first two principal components for each analysis for yield and stay-green traits (maximum NDVI around anthesis (Nmax), indicator for maximum senescence rate (SR), stay-green integral (SGint); thermal time from anthesis to senescence onset (OnS), and mid-senescence (MidS). Each point corresponds to data for a single genotype. The proportion of variation explained by components one and two are given in parentheses. (This figure is available in colour at *JXB* online.)

PCA was used to examine relationships, not only between stay-green traits and yield, but also between the various stay-green traits (Fig. 6). As anticipated, some stay-green traits can be closely correlated, as discussed above, considering the relationships between Nmax, OnS, MidS, and EndS and their influence on SR and SGint.

A close positive relationship was observed between SGint and yield, as the vector for SGint in the first two principal components had similar direction and length to the yield vector in most environments, and almost overlays it in some instances (Fig. 6). Vectors for OnS and MidS were generally in a similar direction to yield, but not as close as SGint, indicating that SGint was more closely related to yield. The exception was in ET2 environments (KTP11rf and GAT11rf), where Nmax was more closely aligned to yield than OnS and MidS, and even SGint in KTP11rf. This supports the high genetic correlations observed between yield and Nmax in these environments (Fig. 4). The Nmax vector aligned poorly with yield in most ETs other than ET2 (except WAR12rf; ET3), and tends to be close to perpendicular to the yield vector in some cases, suggesting that the two are relatively unrelated in such environments. The vector for SR was also generally close to perpendicular with that of yield in all environments, suggesting a weak relationship. The vectors for SR and Nmax were generally pointing in opposite directions, suggesting a negative relationship. This agrees with the fact that a higher SR is required to reach EndS at a similar time after anthesis from a higher Nmax. Accordingly, vectors for SR were generally on the same side of the yield vector as those for OnS, MidS, and SGint, but the opposite side to the Nmax vector. Overall, yield was more closely related to SGint >MidS >OnS >SR >Nmax in all environments except in ET2, where Nmax was more closely related to yield (SGint, Nmax >MidS >OnS >SR).

## Discussion

The study aim was to determine the potential of recently described stay-green traits to improve knowledge about crop adaptation and to select for adaptation to particular TPE.

### Stay-green traits related to whole-of-senescence dynamics are strongly influenced by changes in the water-stress environment

Standardized estimates of component stay-green traits allow comparison of genotype adaptation between different locations and growing seasons. Traits were characterized by fitting a logistic model to NDVI measurements taken at intervals during crop development for each trial plot of each genotype (Christopher *et al.*, 2014), quantifying important characteristics of the stay-green dynamics which differ between genotypes. As discussed above, functional stay-green can be achieved by varying leaf-greenness dynamics in a number of ways (Thomas and Howarth, 2000). Crops may be greener before the onset of senescence (corresponding to higher Nmax), senescence may begin later after anthesis (greater OnS and MidS values), or be slower (lower SR) and/or later finishing (greater EndS) (Thomas and Howarth, 2000; Christopher *et al.*, 2014). Each of these component traits of the stay-green phenotype can contribute to overall green leaf area retention, which is captured by SGint.

Stay-green traits were useful indicators of crop performance in various water-stress environments, despite variation in other important factors affecting yield that could potentially have confounded interpretations including heat stress and radiation limitation. Overall, correlations between stay-green traits and yield were relatively robust and strongly affected by water stress. It is nevertheless important to note that while small differences in phenology were accounted for in the method (using thermal time after flowering), the studied population was also pre-selected to reduce variation for height and anthesis date (Christopher *et al.*, 2013), as these traits are known to affect yield. Some variation in height and anthesis date remained (Fig. 2), but significant correlations with yield occurred in only two of the eight environments for height, and in only one environment for anthesis date.

Overall, Nmax, OnS, MidS, EndS, and SGint all decreased, while SR increased, as water-stress increased from environments in ET1 through to ET4 (Figs 2, 3). All of these traits were highly responsive to water stress.

To enable selection for yield, traits correlated with yield in the relevant environments are preferred.

### Genetic variability for stay-green traits correlates with yield in a broad range of environments including those with little water-stress

Stay-green traits OnS, MidS, and SGint could be used as proxy traits to select for adaptation in a broad range of environments. Stay-green phenotype and related traits have frequently been linked to improved yield in crops experiencing a terminal water stress (in wheat, Christopher *et al.*, 2008; Lopes and Reynolds, 2012; Kipp *et al.*, 2014; in sorghum, Borrell *et al.*, 2000*b*, 2012, 2014*a*, *b*; Jordan *et al.*, 2012; maize, Kamara *et al.*, 2003; Wang *et al.*, 2012; rice, Jiang *et al.*, 2004; Hoang and Kobata, 2009; and a range of crops, Thomas and Smart, 1993; Thomas and Howarth, 2000; Gregersen *et al.*, 2013). Stay-green is also widely recognized as a key drought adaptation mechanism in cereals (Passioura, 2006; Richards, 2006; Cattivelli *et al.*, 2008; Gregersen *et al*, 2013; Thomas and Oughum, 2014). In the absence of water-stress, however, stay-green is not always correlated with yield (in wheat, Lopes and Reynolds, 2012; Gregersen *et al.*, 2013; and in sorghum, Jordan *et al.*, 2012) and can even be associated with reduced yield. For instance, in irrigated wheat and in rice in China, stay-green has been associated with slow export of leaf carbohydrate to the grain, increased lodging, and harvest difficulties due to delayed ripening, all of which can contribute to reduced yield (Gong *et al.*, 2005; Yang and Zhang, 2005). However, even when water is not limiting, increased leaf area duration can lead to prolonged radiation interception and maintenance of photosynthetic capacity, ultimately enhancing potential biomass and grain yield, as shown in sorghum (Borrell *et al.*, 2000*b*). In the current study, the positive correlations between yield and the stay-green traits SGint, OnS, and MidS suggest that in some wheat populations there is little, if any, physiological cost associated with stay-green including under well-watered conditions (ET1). Furthermore, higher values of stay-green traits OnS, MidS, and SGint appear to be beneficial to yield, as illustrated by the positive and consistent correlation with yield in six out of the eight studied environments, including the three least stressed environments GAT10ir, Gat10rf, and WAR11rf (ET1; Fig. 4), as well as a number of water-stressed environments (ET3 and ET4; Fig. 4).

Compared with some other stay-green traits, SR was not as strongly or consistently correlated with yield (Figs 4, 6). For EndS, although higher values were correlated with higher yield across environments (Figs 2h, 3b), EndS was correlated with yield for genotypes within only two environments in the current study. This suggests that EndS is less useful for genetic selection than several other stay-green traits, at least in this genetic material in this TPE using the current method. This result contrasts with results in sorghum, where estimates of senescence late in crop growth are generally highly correlated with grain yield under post-anthesis drought (Borrell *et al.*, 2000*b*, 2014*a*, *b*; Jordan *et al.*, 2012).

Finally, Nmax was found more promising as an indicator of adaptation to intermediate water-stress environments classified as ET2. In ET2, where post-anthesis water-stress was largely relieved later in the season, OnS, MidS, and SGint appeared less related to high yield than in ET1, ET3, and ET4. Thus, for the SeriM82×Hartog population, OnS and MidS could be useful to select for adaptation to either well-watered environments (ET1) or more severely stressed environments (ET3 and 4), but not ET2 environments. In contrast, Nmax appeared more promising in the ET2 environments examined.

Overall, SGint appears the most useful trait to characterize the stay-green phenotype, as it was highly or significantly correlated with yield in all but one of the ET2 environments (KTP11rf; Fig. 4). This is consistent with expectations, since SGint is an integrator of delayed senescence (area under the logistical curve), measuring leaf area duration and hence maintenance of photosynthetic capacity during grain filling.

### Which trait to select where, and how?

SGint had the closest relationship with yield in most environments and will probably be useful for selection in a broad range of environments (Figs 4, 6). However, SGint may be complemented by the quantification of traits such as OnS and MidS, especially in studies looking for physiological processes associated with the stay-green phenotype. Change in SGint can arise from changes in different sets of stay-green traits. For example, a genotype with an earlier onset of senescence (lower OnS) might have a similar SGint to a genotype with later onset, if the rate of senescence is slower and EndS greater (see, for example, fig. 6 in Christopher *et al.*, 2014). In the SeriM82×Hartog population studied, traits associated with delayed onset of senescence and mid-senescence appeared to be the major determinants of SGint and yield adaptation. In conditions where there was little genetic variation in the timing of senescence completion (EndS) between genotypes, this would mean that a higher, rather than a lower, rate of senescence (SR) was associated with higher SGint and higher yield. While higher EndS and lower SR do not appear so useful for adaptation in this study, stay-green due to a slower rate of senescence has been reported in other populations of wheat (Lopez and Reynolds, 2012) and in other species including sorghum (Borrell *et al.*, 2000*a*; Thomas and Howarth, 2000; Harris *et al.*, 2007). Lower SR and greater EndS may prove suitable selection traits in such situations. Similarly, leaf greenness at spike maturity (often measured when the peduncle has senesced) has been proposed as another measure of stay-green associated with improved adaptation in wheat (Lopez and Reynolds, 2012) and sorghum (Borrell *et al.*, 2000*b*). We found little correlation between yield and leaf greenness (NDVI) near the completion of senescence in most environments (Christopher *et al.*, 2014), since in the north eastern Australian TPE, the leaf canopy of wheat is usually fully senesced at the time of spike maturity (Christopher *et al.*, 2008). We did, however, observe an exception at Gatton in 2010 (GAT10ir and GAT10rf), where exceptionally high rainfall and low radiation during the late grain-filling period resulted in the green-leaf area remaining after crop maturity, possibly associated with sink limitation.

The eight environments tested in this study reflect the variability in water-stress environment types (ET1–ET4) encountered across the Australian cropping region in current and future climates, not only in subtropical northern Australia but also in temperate and Mediterranean climatic regions (Chenu *et al.*, 2011, 2013; Watson *et al.*, 2015). The combination of ET1, and the more stressed ET3 and ET4, represents the majority of environments encountered in many regions of Australia (Chenu *et al.*, 2013). Selection for greater SGint, OnS, and MidS should increase adaptation to these environment types. However, these traits did not significantly relate to yield in ET2 (Fig. 4), which occurs less frequently in the TPE. Overall these traits did not have negative effects in any Australian environment types, and appear promising for adaptation of Australian wheats.

Technologies to determine NDVI for a large number of genotypes over the full senescence period are improving rapidly with development of drones and ‘phenomobiles’ (e.g. Chapman *et al.*, 2014; Deery *et al.*, 2014). Measurements of NDVI using a drone to estimate phenology have recently been demonstrated to improve predictions of wheat yield and quality (Magney *et al.*, 2015). Furthermore, there is potential to identify molecular markers that could be used to enrich germplasm for stay-green traits in early generations, before the need for field phenotyping. Thus, the potential to use dynamic stay-green traits to accelerate selection for adaptation to water-stressed environments is predicted to increase rapidly in the near future. We believe that further research using a broader range of genotypes and environments is warranted and would help determine the most suitable stay-green trait(s) for breeding in other environments in internationally important TPE. Further study of the physiological traits underlying changes in stay-green traits is also required to better understand crop senescence and identify associated genetic controls. Comparative genomics will also be helpful, for example, to determine if the stay-green physiological mechanisms (Borrell *et al.*, 2014*a*, *b*) and associated gene networks (Borrell *et al.*, 2015) in sorghum are similar to those in wheat.

### Conclusion

Increased knowledge about the extent of genetic variation in the component stay-green traits will increase our understanding of crop adaptation in relation to water availability. Combining the use of stay-green traits and environmental water-stress characterization, both standardized for thermal time relative to anthesis, provides a powerful method both to characterize, and to select for, adaptation to well-watered and water-stressed environments.

Stay-green traits used singly, or in combination with other traits and/or markers, have great potential for selecting either broad or specific water-stress adaptation. Stay-green traits SGint, OnS, and MidS were positively correlated with high yield in major water-stress environment types encountered in Australian cropping systems, including those environments with little water stress. There appears to be little, if any, yield penalty associated with these traits in any of the tested environments. Nmax could be useful to select for adaptation to moderately post-anthesis stressed environments in TPE where these are important. Overall, these traits have potential to increase the rate of progress towards higher yield with greater yield stability of wheat in a range of environments. The development of molecular markers to select for these traits would be highly desirable, enabling selection in early generations. Understanding the physiology underlying these stay-green traits will also aid in identification of better and more stable markers and/or genes for adaptation to water-stressed environments.

## Supplementary data

Supplementary data are available at *JXB* online.

Figure S1. Yield of wheat cultivar Hartog simulated in the Agricultural Production Systems sIMulator (APSIM-wheat v7) and plotted against adjusted mean yield (best linear unbiased predictors; BLUPs) estimated from measurements at eight trials in south east Queensland during 2010, 2011, and 2012.

Figure S2. Weekly average of daily maximum temperatures and cumulative incident radiation throughout the growing season plotted at dates relative to anthesis of the reference cultivar Hartog for the eight studied environments.

Supplementary Data

## Abbreviations:

^o^Cd: thermal time in degree Celsius days
EndS: thermal time from anthesis to the end of senescence
G×E: genotype by environment interactions
MidS: thermal time from anthesis to midpoint of senescence
NDVI: normalized difference vegetative index
Nmax: maximum NDVI
OnS: thermal time from anthesis to onset of senescence
SGint: senescence integral
SR: senescence rate indicator
TPE: target population of environments.
