# Supplementary material for: Stay-green traits to improve wheat adaptation in well-watered and water-limited environments
Source: J Exp Bot. 2016 Jul 21;67(17):5159–72. doi: 10.1093/jxb/erw276 (PMC5014159; doi:10.1093/jxb/erw276)
Supplement: Supplementary Data [file supp_67_17_5159__index.html]

Stay-green traits to improve wheat adaptation in well-watered and water-limited environments — Stay-green traits to improve wheat adaptation in well-watered and water-limited environments — Supplementary Data 

# Stay-green traits to improve wheat adaptation in well-watered and water-limited environments

## Supplementary Data

Data files

- Supplementary\_figures\_1\_2.pdf - Supplementary Data
