# Supplementary material for: Stay-green traits to improve wheat adaptation in well-watered and water-limited environments
Source: J Exp Bot. 2016 Jul 21;67(17):5159–72. doi: 10.1093/jxb/erw276 (PMC5014159; doi:10.1093/jxb/erw276)
Supplement: Supplementary Data [file supp_erw276_Supplementary_figures_1_2.pdf]

Stay-green traits to improve wheat adaptation in well-watered and water-limited environments

Authors:
Christopher JT, Christopher MJ, Borrell AK, Fletcher S and Chenu K

Supplementary materials

Fig. S1. Yield of wheat cultivar Hartog simulated in the Agricultural Production Systems sIMulator (APSIM-wheat v7) and plotted against adjusted mean yield (best linear unbiased predictors; BLUPs) estimated from measurements at eight trials in south east Queensland during 2010, 2011 and 2012.

Fig. S2. Weekly average of daily maximum temperatures (a) and cumulative incident radiation (b) throughout the growing season plotted at dates relative to anthesis of the reference cultivar Hartog for the eight studied environments. A number of environments in ET2, 3 and 4 experienced heat-stress (a) when weekly average maxima reached or exceeded 30oC near anthesis and the early grain filling period. Frequent rain in trials at Gatton in 2010 (GAT10ir and GAT10rf; ET1) reduced incident radiation compared to other environments (b).

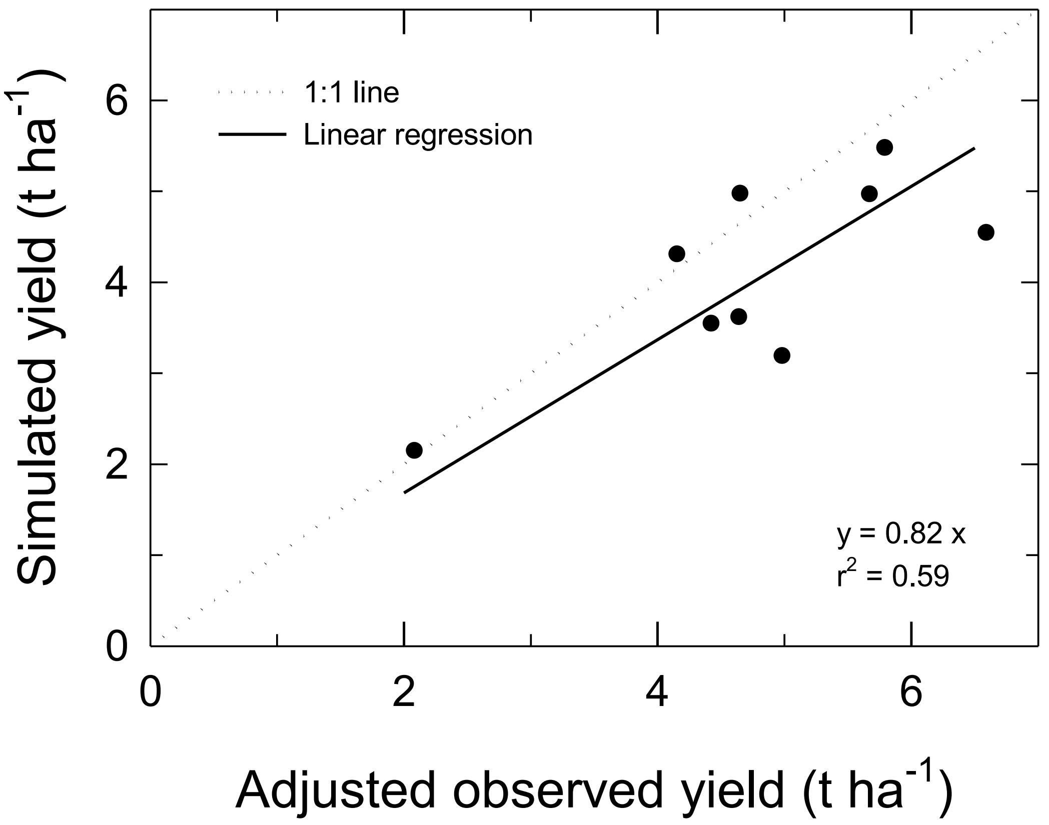
1:1 line
Linear regression
y = 0.82 x
r^2 = 0.59
0
2
4
6
Simulated yield (t ha^{-1})
Adjusted observed yield (t ha^{-1})

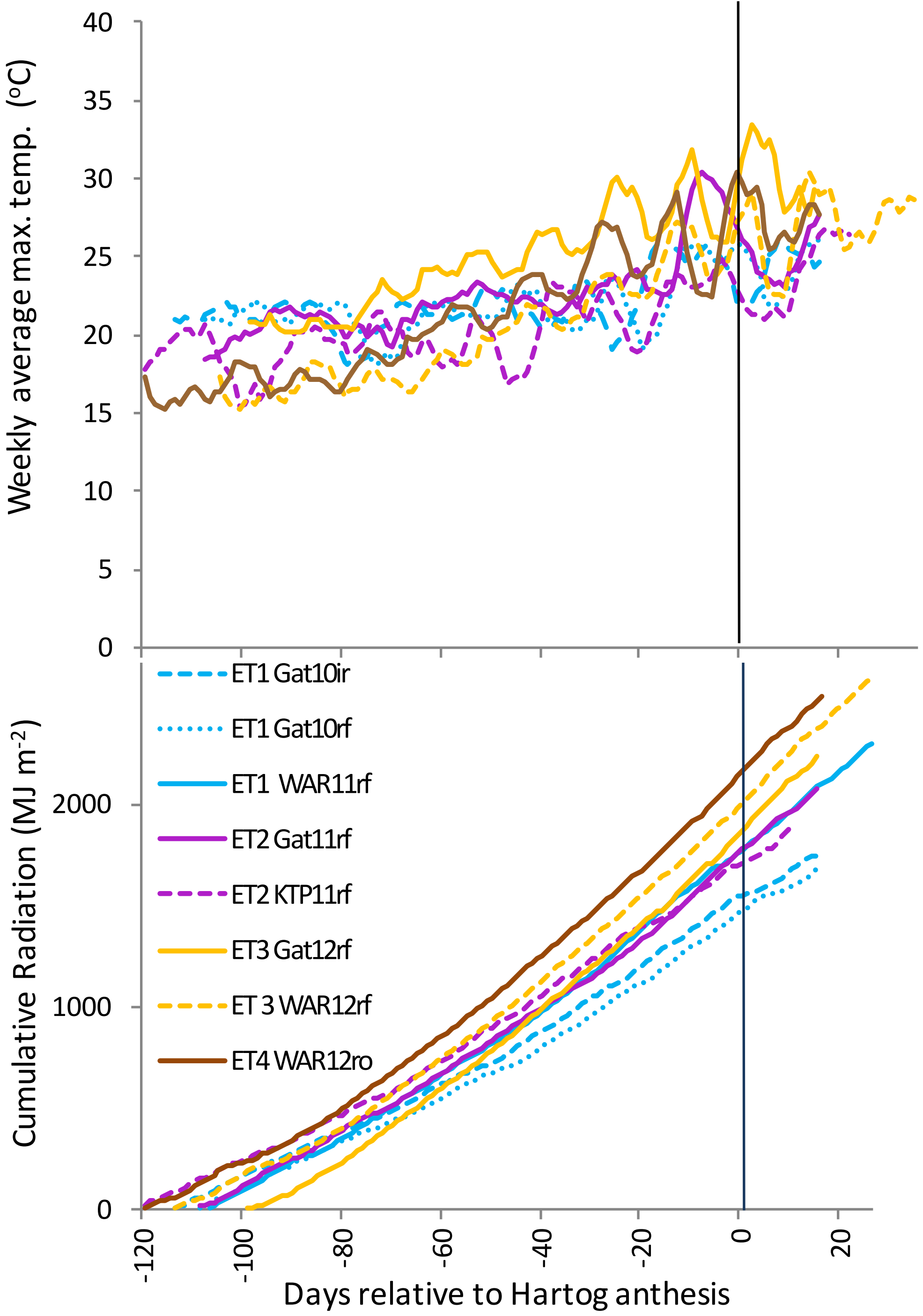
Weekly average max. temp. (°C)
40
35
30
25
20
15
10
5
0
Cumulative Radiation (MJ m-2)
2000
1000
0
ET1 Gat10ir
ET1 Gat10rf
ET1 WAR11rf
ET2 Gat11rf
ET2 KTP11rf
ET3 Gat12rf
ET 3 WAR12rf
ET4 WAR12ro
-120
-100
-80
-60
-40
-20
0
20
Days relative to Hartog anthesis
